# Dipstick for Rapid Diagnosis of *Shigella flexneri* 2a in Stool

**DOI:** 10.1371/journal.pone.0000361

**Published:** 2007-04-18

**Authors:** Faridabano Nato, Armelle Phalipon, Lan Phuong Thi Nguyen, Tai The Diep, Philippe Sansonetti, Yves Germani

**Affiliations:** 1 Plate-Forme 5-Production de Protéines Recombinantes et d'Anticorps, Institut Pasteur, Paris, France; 2 Unité de Pathogénie Microbienne Moléculaire, Institut Pasteur, Paris, France; 3 Unité Institut National de la Santé et de la Recherche Médicale (INSERM) 786, Paris, France; 4 Insitut Pasteur, Ho Chi Minh City, Vietnam; 5 Réseau International, Institut Pasteur, Paris, France; The Research Institute for Children, United States of America

## Abstract

**Background:**

Shigellosis or bacillary dysentery, an acute bloody diarrhoea, is a major public health burden in developing countries. In the absence of prompt and appropriate treatment, the infection is often fatal, particularly in young malnourished children. Here, we describe a new diagnostic test for rapid detection, in stool, at the bedside of patients, of *Shigella flexneri* 2a, the most predominant agent of the endemic form of the disease.

**Methodology/Principal Findings:**

The test is based on the detection of *S.flexneri* 2a lipopolysaccharide (LPS) using serotype 2a-specific monoclonal antibodies coupled to gold particles and displayed on one-step immunochromatographic dipstick. A concentration as low as 20 ng/ml of LPS is detected in distilled water and in reconstituted stools in under 15 minutes. The threshold of detection corresponds to a concentration of 5×10^7^ CFU/ml of *S. flexneri* 2a, which provides an unequivocal positive reaction in three minutes in distilled water and reconstituted stools. The specificity is 100% when tested with a battery of *Shigella* and unrelated strains, in culture. When tested in Vietnam, on clinical samples, the specificity and sensitivity were 99.2 and 91.5%, respectively. A decrease of the sensitivity during the evaluation on stool samples was observed after five weeks at room temperature and was due to moistening of the dipsticks caused by the humidity of the air during the fifth week of the evaluation. This drawback is now overcome by improving the packaging and providing dipsticks individually wrapped in waterproof bags.

**Conclusion:**

This simple dipstick-bases test represents a powerful tool for case management and epidemiological surveys.

## Introduction

Shigellosis, an acute bloody diarrhea caused by the Gram negative entero-invasive bacterium *Shigella* spp, represents a major public health burden in many developing countries [1]. According to a reference study published in 1999, which provided projections derived from literature-based data [2], the annual number of *Shigella* episodes throughout the world was estimated to be about 164.7 million, with 99% occurring in developing countries. The estimated number of deaths is about 1.1 million. Children under the age of 5 are the main target of the disease, representing 69% of all episodes and 61% of all deaths. Although *S. dysenteriae* type 1 is associated with the most severe form of the disease and high mortality rate when epidemics occur, most of the deaths are attributable to the endemic form of the disease, which is most often caused by *S. flexneri*. In the reference study cited above, the median percentages of isolates of *S. flexneri, S. sonnei, S. boydii*, and *S. dysenteriae* were 60%, 15%, 6%, and 6% (with 30% of *S. dysenteriae* isolates being serotype 1) in developing countries, and 16%, 77%, 2%, and 1% in industrialized countries, respectively. In developing countries, the predominant *S. flexneri* serotype is 2a [2].

A recent study by Kosek et al [3] indicated that the incidence of diarrhoeal diseases remains stable but their mortality rate is tending to decrease. Is *Shigella* following this tendancy? With regard to mortality, it has indeed very significantly decreased. The main reasons are likely to be the lack of a major *S. dysenteriae* 1 outbreak for at least 10 years, improvement of mothers' education, better primary care, better nutrition status of children in economically emerging Asian countries, and presumably the large and uncontrolled use of antibiotics. Nevertheless, the steady increase of antibiotic resistance makes the emergence of massive epidemics of *S. dysenteriae* 1 a possible scenario, particularly in socially unstable areas. Indeed, isolates are now largely resistant to first line antibiotics (ampicillin, chloramphenicol, tetracycline, sulfamides, trimethoprime plus sulfamides, nalidixic acid) and resistance to second line antibiotics including fluoroquinolones becomes increasingly prevalent [1], [4], [5], [6], [7].

With regard to morbidity, a recent multicentric study carried out in six Asian countries [5] showed that *Shigella* is isolated in at least 5% of the cases of diarrhoea, a remarkably stable value. However, it seems that the incidence of shigellosis is largely underestimated. A major reason is the weakly fastidious nature of *Shigella* which poorly survives transport, and for which no enrichment medium exists. The traditional identification by culture lacks sensitivity due to the low number of causative micro-organisms, competition with commensal organisms, and deleterious changes in ambient temperature and pH during specimen transport [8], [9], [10]. The detection is also frequently impaired by the use of antibiotics prior to specimen collection. Consequently, only a fraction of the current cases of shigellosis are presumably detected [9]. This was confirmed in a recent study showing that prospective studies using optimized procedures of collection and rapid processing yield incidence data that are several folds higher than those obtained by passive collection of data in natural health cohorts [**4**].

There is therefore a need for updated epidemiological data, particularly in Africa where the pattern of diarrhoeal diseases seems generally more severe, in terms of both morbidity and mortality [11], [12], [13]. For such studies, improved diagnostic tools are needed to complete the currently used classical microbiological methods. In particular, improvement of the methods of enrichment and isolation and novel diagnostic tools are required. They should be robust, quick, reliable (sensitive and specific), efficient on fecal samples and easy to use at patient's bedside. Two approaches have emerged: PCR detection [9], [5], [14], [15], [16], [17] and immunochromatographic techniques, *i.e.* dipsticks based on the recognition of pathogen-specific antigens by monoclonal antibodies (mAbs). Such dipsticks have already been successfully developed for cholera [18], meningitidis [19] and plague [20]. In this study, we investigated the potential of such dipsticks to detect *S. flexneri* 2a that remains the most frequently isolated serotype in endemic areas. The dipstick is based on the detection of lipopolysaccharide (LPS), the major bacterial surface antigen. Indeed, *Shigella* serotypes are defined by the structure of the oligosaccharide repeating unit (RU) that forms the O-antigen (O-Ag), the polysaccharide moiety of LPS [21]. For *S. flexneri* serotype 2a, the biological RU is a branched pentasaccharide. It is composed of a linear tetrasaccharide backbone made of three L-rhamnose residues, **A, B**, and **C**, and a *N*-acetyl-D-glucosamine residue **D**, that is common to all *S. flexneri*, except serotype 6, and of an α-D-glucose residue **E**, branched at position 4 of rhamnose **C** that specifies serotype 2a [21], [22].

**Figure 1:**
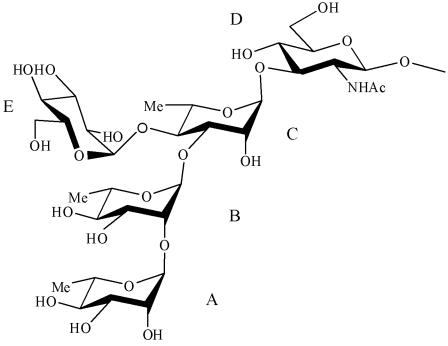
Structure of the repeating unit of the O-Ag of *Shigella flexneri* serotype 2a.

In this study, we demonstrate that a dipstick based on mAbs recognizing serotype 2a-specific determinants carried by the LPS O-Ag (Figure 1) is a rapid, robust and reliable test to identify *S.flexneri 2a* in stool samples.

## Materials and Methods

### Development of *S. flexneri* 2a dipstick

To develop mAbs against the somatic antigen of *S. flexneri* 2a [22], BALB/c mice were immunized intraperitonally (i.p.) with 10^7^ CFU of killed *S. flexneri* 2a bacteria, three times at 3-week intervals. Mice eliciting the highest anti-LPS antibody response were given an intraveinous boost injection 3 days before being sacrificed for splenic B cell fusion, according to Kohler and Milstein [23]. Hybridoma culture supernatants were screened for antibody (Ab) production by ELISA using LPS purified from *S. flexneri* serotype X, Y, 5a, 5b, 2a, 2b, 1a, and 3a, respectively, as previously described [24], [25]. Briefly, LPS purified according to Westphal and Jann [26] was used at a concentration of 5 µg/ml in PBS. As secondary Abs, anti-mouse IgG-, IgM-, or IgA-alkaline phosphatase-labeled conjugate (Sigma-Aldrich) were used at a dilution of 1/5,000. Only the hybridoma cells secreting mIgG reacting specifically with LPS homologous to the strain used for immunization, *i.e*., recognizing serotype-specific determinants on the LPS O-Ag, were selected. The selected hybridomas, representative of the four murine IgG subclasses, were then cloned by limiting dilution, and injected i.p. into histocompatible mice for ascite production. mIgG were precipitated with 50% ammonium sulfate from ascite fluid, centrifuged, and dialyzed against PBS before being purified using ion-exchange chromatography as previously described [24], [25].

Among the available mAbs specific for *S. flexneri* serotype 2a, two IgG2a were selected for the development of the diagnostic test. The test is based on a one-step, vertical-flow immunochromatography using mAb-coupled colloidal gold particles [27]. The colloidal gold particles (40 nm diameter) were conjugated to the D15-7 anti-*S. flexneri* 2a mAb (British Biocell International Cardiff, UK) and lyophilised (A_540_nm = 2) onto polyester release pads (Accuflow P Schleicher&Shull, Mantes la Ville, France). An automatic thin layer chromatography sampler (CAMAG 5, Muttenz, Switzerland) was used to spray the second selected anti-*S.flexneri* 2a mAb, E4-1, at a concentration of 2 µg/cm, as a line on nitrocellulose membrane (Immunopore FP, Whatmann International). In addition, a control capture line was obtained by spraying affinity-purified goat anti-mouse IgG (ICN Biomedical, Aurora, Ohio, USA), on a line higher up on the strip, at a concentration of 1 µg/cm. Cellulose filter paper was used for the wicking and sample pads (Cellulose paper 903, Schleicher&Shull).

**Figure 2:**
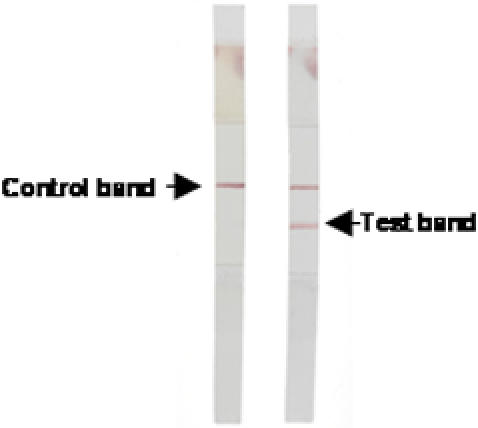
Two dipsticks showing typical negative and positive results after being kept for 15 min in diarrhoea stool samples.

The immunostrips were then trimmed to a width of 5 mm (Figure 2) and stored in a waterproof bag (50 per bag) at room temperature in Paris (France) or sent to Ho Chi Minh City (Vietnam) to be evaluated. The test was carried out in 5 ml-disposable plastic tubes at room temperature with a sample volume of about 300 µl. After 5–15 min, a positive result appears as two pink lines (upper control line and lower *S. flexneri* 2a LPS-positive line), and a negative result as a single upper pink control line (Figure 2). *S. flexneri* strain 2a (ref. NCDC 2747-71) was used as positive control.

### Methodology of the *S. flexneri* 2a dipstick evaluation

The sensitivity and the specificity of the assays were assessed by one trained technician. The cut-off (detection limit) and the range of detectable LPS concentrations was measured using tenfold dilutions (1 µg/ml to 10 ng/ml) of purified LPS 2a and tenfold dilutions of a *S. flexneri* 2a suspensions (5×10^8^ to 5×10^3^ bacteria/ml) using saline, and reconstituted stools (10 g of normal stool without *Shigella* spp suspended in 10 ml of saline). Reliability of immunostrips was assessed with purified LPS (1 µg/ml). To predict the shelf-life of the dipstick, we used the accelerated stability method that consisted in storing the assays for a time at elevated temperature [27]. The shelf life of the strips in the laboratory was assessed by testing three times per week for 10 weeks after storage at 25°C (air-conditioned room) or at 60°C (incubator).

The specificity was assessed using pure cultures of *S. flexneri* (serotypes 1a, 1b, 2a, 2b, 3a, 3b, 4a, 4b, 5a, 5b, 6, Y, X), *S. dysenteriae* (serotypes 1-15, and untypable strain 97-10607), *S. boydii* (serotypes 1 to 20), *S. sonnei* (strains in phase I, phase II), *Salmonella enterica typhimurium* (strains 06-2835, 06-2846, 06-2847), *S enteritidis* (strains 06-2841, 06-2844, 06-2851, 06-2852), *S. hadar* (strains 06-2533), *S. brandenburg* (strain 06-2619), *S. heidelberg* (06-2843), *S. oranienburg* (strain 06-2634), *S. risen* (strain 06-2615), *S. stanleyville* (strain 06-2832), *S. typhi* (strain 06-2829), *S. paratyphi* A (strain 06-2633), *S. paratyphi* B (strain 06-2696), *S. meleagridis* (strain 06-2850), *S. stubra* (strain 06-2384), *S. huittingfoss* (strain 06-2391), enteroagregative *Escherichia coli* (strains 55989, JM221, O42, 56390 and 384P), diffusely adherent *E. coli* (strain AL851, AL847, C1845, AL855 and 3043), enterotoxigenic *E. coli* (strains EDL1496, 440TL, Tx-1, E2539-C_1_, 469), enteropathogenic *E. coli* (strains 135/12 (O55:H-), E6468/62 (O86:H34), 11201 (O125:H6), KK111/1 and F88/6848-2 both O26:H11), *Vibrio cholerae* O1 (strains CNRVC960255, 970002, 970014, 970025, 970067, 960325, 970022, 970053, 970055, 970056), *V cholerae* O139 (strains CNRVC 930008, 930381, 930210, 930190), *V. cholerae* non O1 and non O139 (strains CNRVC 930177, 930429, 950689, 950691, 970037, 950769, 910388, 930121, 930297, 930391), *V. alginolyticus* (strain CIP103336), *V. fluvialis* (strains CIP103355, CNRVC356), *V. parahaemolyticus* (strains CIP75.2, CNRVC030478, CNRVC030479, CNRVC000204, CNRVC000208), *V. furnissii* (strain CIP102972), *V. hollisae* (strain CIP104354), *V. mimicus* (strain 101888), *Aeromonas caviae* (strain CIP76.16), *A. enteropelogenes* (strain CIP104434), *A. hydrophila* (strain CIP76.15), *A. sobria* (strain CIP74.33), *Plesiomonas shigelloides* (strain CIP63.5), *Campylobacter jejuni, Yersinia enterocolitica* 1A (6 strains of biotype 1A, 2 strains of biotype 2, 2 strains of biotype 3, and 2 strains of biotype I).

Sensitivity and specificity of the dipsticks were evaluated in a clinical setting in Ho Chi Minh City, an area of dysentery endemicity, during a period of high incidence of the disease [5]. The study has been approved by the Scientific and Ethical Committee of Pasteur Institute in Ho Chi Minh City. The dipsticks were shipped from France to Vietnam at ambient temperature in grip seal bags by air mail. We compared the results obtained with stool cultures for enteropathogenic bacteria and dipsticks performed in a blind study by two different technicians. A total of 191 stool samples from infants hospitalized for dysentery were collected and tested in the Paediatric Hospitals I and II at Ho Chi Minh City. The dipstick test was performed on freshly collected stools. A volume of about 200 µl of stools (preferentially blood, mucus, rectal sputum when present) were transferred with a disposable pipette in a haemolysis glass tube of 5 ml containing 300 µl of distilled water. The sample was homogenised by several pipetting. The immunostrip was then introduced in the test tube and the test was read after 15 min.

Stool samples were transported to the microbiology laboratory of the Pasteur Institute of Ho Chi Minh City for diagnosis by classical methods. In brief, stool samples were cultured for *Shigella* spp and other enteric pathogens on Hektoen enteric agar and Bromocresol Purple Lactose agar [28]. Suspected colonies resembling *Shigella* were identified biochemically and serotyped. *S. flexneri* 2a colonies were identified by slide agglutination with anti-II and anti-3,(4) sera (Eurobio, France), according to the International *Enterobacteriaceae* Grouping Subcommittee [29].

Samples that were positive by the dipsticks but negative by culture were stored at −20°C and later examined by a PCR targeting the *ipaH* gene of *Shigella* spp [30], [31].

## Results

The objective of the study was to develop and evaluate a dipstick test for the rapid diagnosis of *S. flexneri* 2a infection using stool samples at the bedside of the patient. The evaluation was performed firstly by using purified LPS of *S. flexneri* 2a in distilled water and then in reconstituted stools at different concentrations. The dipsticks were then tested on various concentrations of *S. flexneri* 2a in culture and in reconstituted stools. The dipsticks were also tested with various species of bacteria in cultures, and finally on diarrhoeal stools.

In distilled water, the lower detection threshold of the dipstick for *S. flexneri* 2a was 20 ng/ml of LPS. The same threshold was observed in stools reconstituted with different amount of purified *S. flexneri* 2a LPS. In addition, both in distilled water and in reconstituted stools containing different concentrations of *S. flexneri* 2a, an unequivocal positive reaction was obtained in 10 minutes with 5×10^7^ CFU/ml of *S. flexneri* 2a. The specificity of the dipstick was 100% for all bacterial cultures. Similar results were obtained using dipsticks stored for 7 days at 60°C and 10 weeks at 25°C. No prozone effect (*i. e*. no signal detected for high concentrations) was observed by using a range of LPS concentrations extending from 10 ng/ml to 1 µg/ml.

**Figure 3:**
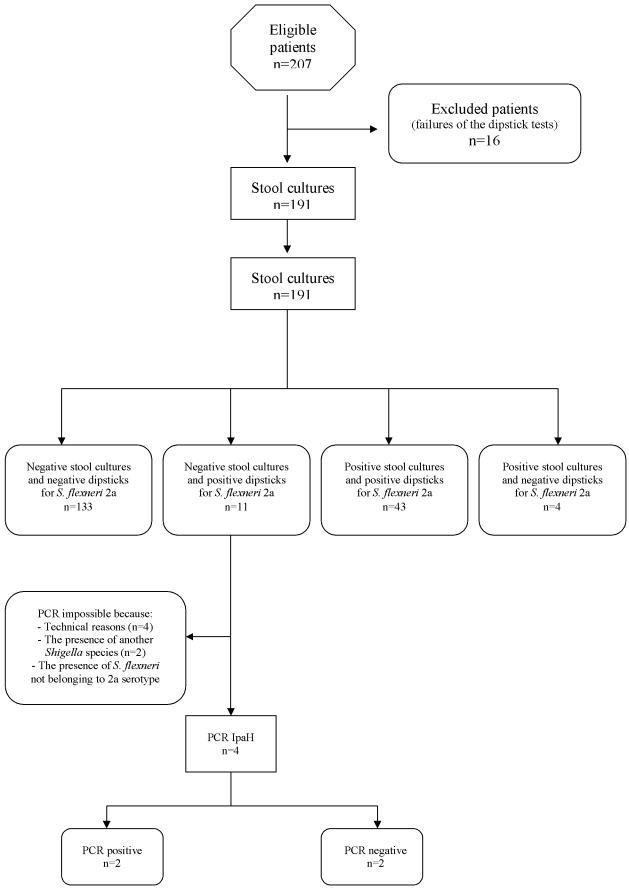
STARD flow diagram of the evaluation study in Vietnam.

Of the 191 stool samples from patients displaying symptoms of dysentery (Figure 3), 43 were both dipstick-and culture-positive, 11 were dipstick-positive but culture-negative, 4 were dipstick-negative but culture-positive, and 133 were negative by both culture and dipstick (Table 1). Specificity (133/144) on the field was therefore 99.2% (95% CI 97.8%–100%), the sensitivity (43/47) was 91.5% (95% CI 83.6%–99.4%), positive predictive value (43/54) was 79.6% (95% CI 69%–90.3%) and negative predictive value (133/137) 97% (95% CI 89.6%–100%).

**Table 1:** Detection of *S. flexneri* 2a in 191 fresh stool samples by dipstick test versus conventional culture.

Bacteriological culture	N° of specimens with *S. flexneri* 2a dipstick test result		
	Positive	Negative	Total
Positive	43	4	47
Negative	11	133	144
Total	54	137	191

Among the 11 samples which were positive by the dipsticks, but negative by bacteriological culture for *S. flexneri* 2a, *S. sonnei* was isolated from 2 patients and *S. flexneri* not belonging to the 2a serogroup was isolated from one patients. For these three cases, it was not possible to conclude if there were co-infections because no PCR specific for *S. flexneri* 2a was available. In one sample, *Salmonella enterica* was isolated; the *ipaH* PCR was positive and the patient was presumably co-infected with *S. flexneri* 2a. In two samples, the PCR was negative; the presence of PCR inhibitors in these stool samples was suspected, but false-positive results with the dipsticks cannot be excluded. One sample was *ipaH* PCR positive; the prior use of antibiotics by this patient before the sampling was documented and may have lessened the excretion of viable *S. flexneri* 2a in the stool sample. For technical reasons (insufficient amount of stools, problems of storage), PCR was not done on four samples.

The four stool samples which were negative by the dipstick test were positive for *S. flexneri* 2a by bacteriological culture. One of the reasons for such a result might be that the amount of LPS in these stool samples was lower than the threshold level. Another explanation could be the decrease of the sensitivity of the conjugated mAb due to moisture, because these dipsticks were used after a delay of five weeks at ambient temperature during the rainy season. Positive controls worked well but appeared weaker than previously, even when the test was carried out with pure culture of *S. flexneri* 2a (at least 5×10^8^ CFU/ml).

The blindness of the study was broken to control the serotype of all the strains of *Shigella flexneri* in case of discordant results; all the serotypes were confirmed after the second serogrouping.

From the 133 dipstick-and culture-negative samples, 21 cultures were positive for *S. enterica* (including one co-infection with *S. sonnei*), 20 for *S. sonnei*, 1 for *S. dysenteriae* 2, and 9 for *S. flexneri* not belonging to serotype 2a.

## Discussion

Severe and milder forms of shigellosis are developed by patients living in endemic areas. Dysenteric patients have a more severe form of shigellosis with a clinical spectrum ranging from watery diarrhea to diarrhea with mucus and frank bloody diarrhea. Bloody diarrhea is associated with the rupture of the intestinal epithelial barrier, followed by the invasion and destruction of the intestinal mucosa, resulting in the proliferation of the pathogens faster than that occuring in patients with a milder form of the disease [32]. Patients who have the most severe form of shigellosis also shed an higher number of micro organisms [32]. A direct relationship between bacterial load (*i. e*. LPS concentration in stools), detection by culture, and disease severity has also been reported by Thiem et al [9]. Consequently, it is essential to develop an efficient dipstick test displaying a low detection threshold, and detecting the somatic antigen without prozone effect to avoid false-negative results in samples containing high concentrations of *S. flexneri* 2a LPS antigen.

We report here such a tool. The *S. flexneri* 2a dipstick we developed was found to be highly specific when tested on bacterial cultures, with a detection threshold comparable to those of dipsticks developed to diagnose cholera (10^7^ CFU/ml of *V. cholerae* O1 and 50 ng/ml of LPS) [18], [33]. The *S. flexneri* 2a dipstick detected somatic antigen at a wide range of concentrations, in 5–15 minutes, without prozone effect.

The evaluation on stool samples of patients living in endemic area confirms the good specificity of the *S. flexneri* 2a dipstick test. Regarding the sensitivity of 91.5% measured during the “on field evaluation” as compared to that using laboratory testing, the most likely explanation for the discrepancies observed for four samples that appeared false negative by *S. flexneri* 2a dipstick is certainly the condition in which the dipsticks were preserved locally. This problem of decreasing of sensitivity due to humidity was also observed with other dipsticks developed for other pathogens [20]. This drawback is now overcome by improvement of the individual dipstick packaging, making them easily transportable and adapted to the local environmental conditions. Although the study is not completed, we already have new data with two other evaluations that are undergoing in Santiago (Chile) and Djibouti (with the French Army) by using dipsticks individually wrapped in waterproof bags. The results we obtained demonstrate that the problem has been resolved. After about 5 weeks of storage of the dispsticks at room temperature, the tests have been performed on positive and negative stools stored at −20°C in Santiago. We observed that the concordance was 100% (data not shown). Another evaluation is currently being performed in Dhaka with a special attention to this problem.

The test was applied to the diagnosis of bacillary dysentery to watery stools, frank bloody stools, as well as stools with mucus and rectal mucous discharge. Although the diarrheic stools contain high concentrations of *S. flexneri* 2a somatic antigen in the severe forms of the illness (*i.e*. true dysenteric syndrome), we thought that it was important to dilute the sample in distilled water to increase antigen-antibody interactions and to improve the flow of immune complexes through the dipstick. Otherwise these could be inhibited by the viscosity and the density of the rectal sputum or of the mucus.

The reference test–isolation, biochemical and seroagglutination of *Shigella*–which can be done only in the laboratory is specific but lacks sensitivity. This may explain why *S. flexneri* 2a was missed in eleven stools that were dipstick-positive. Co-infections involving probably *S. flexneri* 2a and other enteropathogenic bacteria were observed in 4 samples (2 with *S. sonnei*, 1 with *S. flexneri* not belonging to serogroup 2a, and 1 with *S. enterica*). This is explained by the fact that during routine work, only five lactose negative colonies were studied per stool, a condition which is not in favour of the diagnosis of infections with multiple enteropathogenic bacteria. Indeed the coproculture is generally stopped when an enteric bacterium in relation to the clinical symptoms is isolated, but enteric infections with multiple pathogens are frequent in developing countries in endemic tropical area [28]. Reasons for the low sensitivity of traditional culture methods also include the low number of causative *Shigella* strains in several cases, competition from other commensal microorganisms, and inappropriate changes in ambient temperature and pH during specimen transport [10]. The growth, and thus the detection, of the bacteria is further impaired by the use of antibiotics prior to specimen collection (one documented case in this study). However, the coproculture remains “indispensable” to complete the diagnosis in particular for determining antibioresistance and for characterization of the strains.

No specific PCR was available to confirm the presence of *S. flexneri* 2a in the case of negative results of the coproculture, but two infections with *S. flexneri* 2a (including one co-infection with *S. enterica*) were highly suspected because the dipsticks and the PCR assay based on the amplification of the invasion plasmid antigen H (*ipaH*) gene sequence were positive.

Because prompt diagnosis of diarrhoea is of key importance to initiate effective therapy and to decide proper epidemiological measures, multivalent dipsticks are needed. Therefore, we are currently developing other dipsticks for *Shigella* spp (generic diagnosis) and the most prevalent serotypes (*S. dysenteriae* 1, 2 and 3; *S. flexneri* 1b, 2b, 3a, 6b; *S. sonnei*), but also for *Salmonella enterica*, diarrheogenic *Escherichia coli* (EIEC, EPEC, EHEC, EAEC), *Campylobacter spp, E. histolytica, Giardia lamblia*. These tools will complete those actually available for Rotavirus and *V. cholerae* O1 and O139.The use of PCR assay based on the amplification of the invasion plasmid antigen H (*ipaH*) gene sequence can overcome some of the shortcomings of culture methods, but the method itself has not yet received global acceptance due to difficulties in the implementation by some laboratories located in developing countries.

In this study, we demonstrated the proof of principle of a one-step, vertical-flow immunochromatography test based upon mAbs recognizing serotype-specific determinants carried by the O-Ag as a rapid, robust and reliable test to identify *S. flexneri* 2a from stool samples. This new diagnosis test, which requires minimal technical skill efficiently completes the classical microbiological methods.

The importance of an efficient bacillary dysentery surveillance system continues to be stressed by World Health Organization with regard to improving risk assessment of potential bacillary dysentery outbreaks [1]. To fullfill this need, further studies are under way to develop: i) a single test able to diagnose all *Shigella* spp (whatever the serotype) and EIEC, ii) a multiplex dipstick for several prevalent serotypes of *Shigella* spp (*ie. S. dysenteriae* 1, *S. flexneri, S. sonnei*, etc.) that will be evaluated in different settings in endemic countries including: on the field, in dispensary (remote area), and in public health laboratories.
